# A multimodal endoscopic approach to early cardiac cancer in gastritis cystica profunda

**DOI:** 10.1055/a-2796-7617

**Published:** 2026-03-09

**Authors:** Yujie Zhu, Ning Wang, Lingyun Wang

**Affiliations:** 1 Jining Medical University, Jining, China; 2 Department of Gastroenterology, Yutai County Peopleʼs Hospital, Jining, China; 3 117947 Department of Gastroenterology, Jining No. 1 Peopleʼs Hospital, Jining, China

A 68-year-old male patient presented with dysphagia. ( Fig. 1 ). Biopsy pathology indicated “high-grade dysplasia with malignant transformation.” Computed tomographic images showed thickening of the cardia wall, mild non-uniform enhancement on contrast-enhanced scanning, poor filling of the remaining stomach, and thickened walls ( Fig. 2 ). To further evaluate the nature and depth of the lesion, the patient underwent endoscopic ultrasonography (EUS), which revealed “multiple anechoic structures at the lesion site, confined to the submucosa, with unclear boundaries between the mucosal and muscular layers, partial section 8.5 mm × 10.6 mm, and a high likelihood of deep cystic gastritis (GCP; Video 1 ).” This first identified the soil for malignant transformation. An endoscopic submucosal dissection (ESD) procedure was performed for perfect specimen removal ( Fig. 3 ). Postoperative histological sections clearly showed the coexistence of submucosal infiltrating adenocarcinoma ( Fig. 4 **a** ) and benign cystic glands of gastritis cystica profunda (GCP; Fig. 4 **b** ). This further validated the EUS diagnosis. Immunohistochemical results showed P53 expression in a mutated pattern ( Fig. 5 **a** ). The Ki67 proliferation index was approximately 40% ( Fig. 5 **b** ), and desmin staining clearly demonstrated invasion into the muscularis mucosae and vasculature ( Fig. 5 **c** ).

**Fig. 1:**
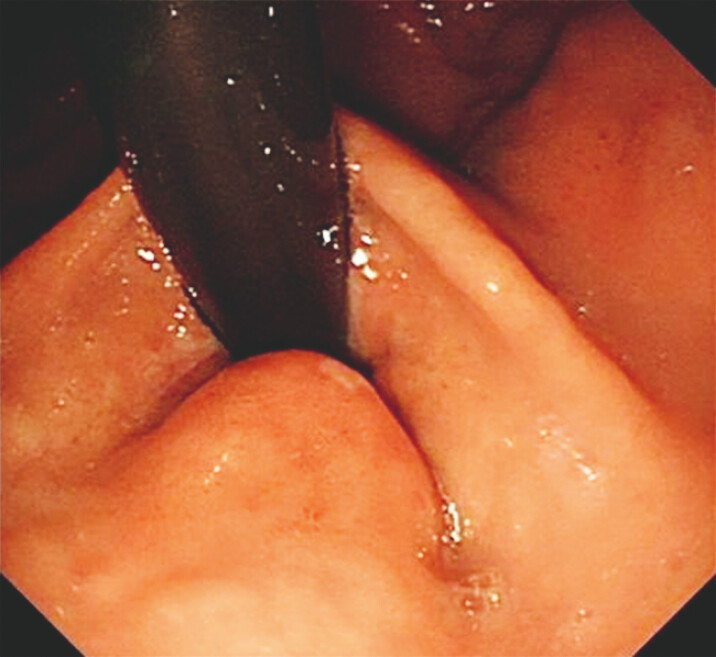
A raised area of about 1.5 cm in diameter on the lesser curvature of the cardia, congested, with surface ulceration.

**Fig. 2:**
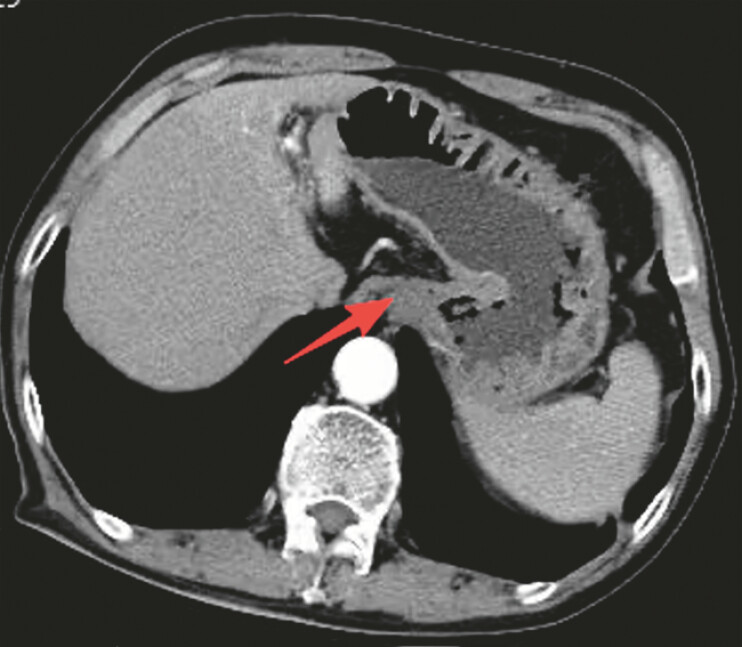
Thickening of the cardia wall, mild non-uniform enhancement on contrast-enhanced scanning, poor filling of the remaining stomach, and thickened walls.

Multiple anechoic structures at the lesion site, confined to the submucosa, with unclear boundaries between the mucosal and muscular layers, partial section 8.5 mm × 10.6 mm, and a high likelihood of deep cystic gastritis (GCP).Video 1

**Fig. 3:**
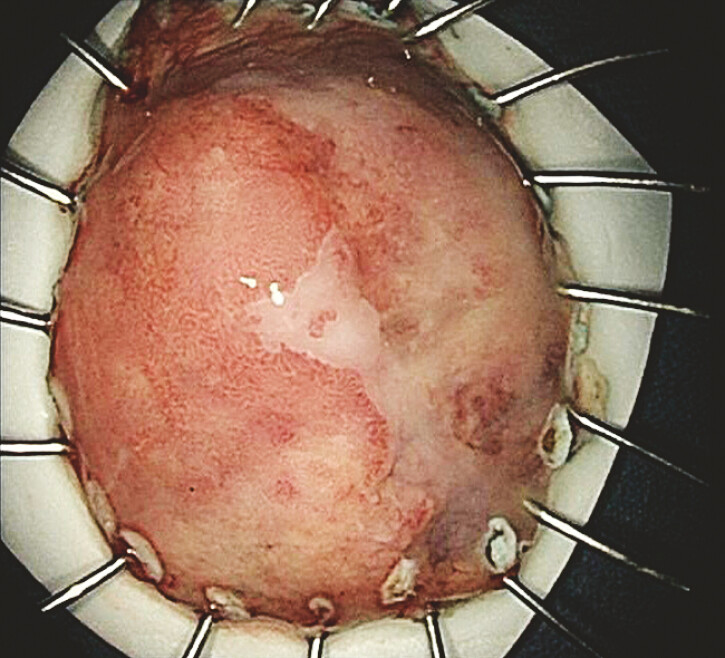
Complete resection specimen, size of about 4.5 × 3.5 × 0.2 cm, surface of a rise, size of about 2.5 × 2 × 0.8 cm, according to the surrounding cut edge of 0.4–1.4cm.

**Fig. 4:**
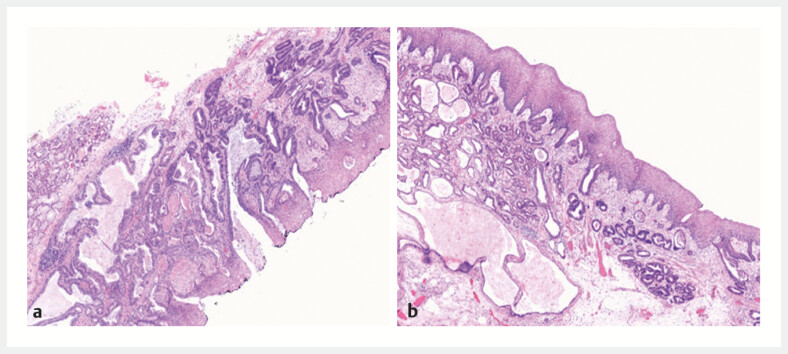
**a** Paraglandular adenomatous hyperplasia (magnification × 40). **b** Deep cystic gastritis of the local mucosa (magnification × 40).

**Fig. 5:**
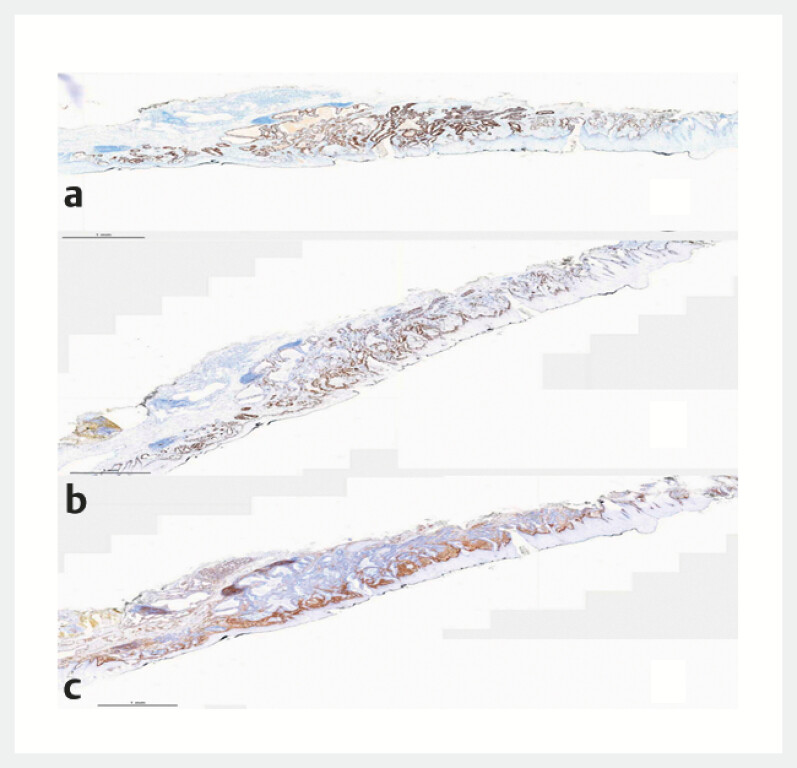
**a** P53 expression in a mutated pattern, supporting the diagnosis of highly dysplastic hyperplasia. **b** The Ki67 proliferation index was approximately 40%. **c** Desmin staining clearly demonstrated invasion into the muscularis mucosae and vasculature.

GCP is characterized by the ectopic extension of gastric mucosal glands into the submucosa, accompanied by cystic dilation. The lesion most commonly occurs in the gastric cardia and subcardinal regions, predominantly affecting elderly men. 1 Although GCP itself is often considered benign, accumulating evidence indicates a close association between GCP and early gastric cancer, particularly high-grade intraepithelial neoplasia 2 3 . This case corroborates the existing literature consensus that GCP represents a significant precancerous condition and suggests its potential for malignant transformation 4 5 . This video demonstrates a comprehensive multimodal strategy, combining endoscopic ultrasound (EUS), cross-sectional imaging, and histopathology with immunohistochemistry (IHC) to guide the endoscopic resection of an early cardiac carcinoma arising from GCP.

Endoscopy_UCTN_Code_CCL_1AB_2AC_3AB

## References

[1] Yuxin J Clinical characteristics of deep cystic gastritis: a summary analysis with 9 case reports Dalian Dalian Medical University 2022

[2] Ting Z Jianbo Z Fangfang Z A case of gastric cancer complicated with deep-seated cystic gastritis J Wenzhou Med Univ 2022 52 325 327

[3] Hui F Pengyue Z Yongping C Clinical analysis of 64 cases of deep-seated cystic gastritis complicated with early gastric cancer Anhui Med J 2023 44 1086 1090

[4] Fuping G Junrong Y Jin W Clinical and pathological characteristics of 13 cases of deep-seated cystic gastritis complicated with early gastric cancer Anhui Med J 2022 26 528 530

[5] Niu N Endoscopic and pathological features of deep-seated cystic gastritis complicated with early gastric cancer J Clin Oncol 2023 28 1046 1049

